# Synthesizing a Cellulase like Chimeric Protein by Recombinant Molecular Biology Techniques

**DOI:** 10.4172/2155-9821.1000285

**Published:** 2016-06-30

**Authors:** Hirendra Nath Banerjee, Christopher Krauss, Valerie Smith, Kelly Mahaffey, Ava Boston

**Affiliations:** Department of Natural Pharmacy and Health Sciences, Elizabeth City State University, University of North Carolina, Elizabeth City, NC-27909, USA

## Abstract

In order to meet the Renewable Fuels Standard demands for 30 billion gallons of biofuels by the end of 2020, new technologies for generation of cellulosic ethanol must be exploited. Breaking down cellulose by cellulase enzyme is very important for this purpose but this is not thermostable and degrades at higher temperatures in bioreactors. Towards creation of a more ecologically friendly method of rendering bioethanol from cellulosic waste, we attempted to produce recombinant higher temperature resistant cellulases for use in bioreactors. The project involved molecular cloning of genes for cellulose-degrading enzymes based on bacterial source, expressing the recombinant proteins in *E. coli* and optimizing enzymatic activity. We were able to generate *in vitro* bacterial expression systems to produce recombinant His-tag purified protein which showed cellulase like activity.

## Introduction

Cheap, clean, green energy production is a goal of Department of energy and EPA. Biofuels are made by converting renewable materials--for example, corn kernels, wood chips left over from pulp and paper production, prairie grasses, and even garbage--into fuels and chemicals. Most biofuels used today are made from the fermentation of starch from corn kernels. That process, although simple, is costly because of the high price of the corn kernels themselves.

Agricultural waste, such as corn stover (the leaves, stalks, and stripped cobs of corn plants, left over after harvest), is cheap. These materials are largely composed of cellulose, the chief component of plant-cell walls. Cellulose is far tougher to break down than starch. An additional complication is that while the fermentation reaction that breaks down corn starch needs just one enzyme, the degradation of cellulose requires a whole suite of enzymes, or cellulases, working in concert.

The cellulases currently used industrially, all of which were isolated from various species of plant-decaying filamentous fungi, are both slow and unstable, and, as a result, the process remains prohibitively expensive. Even a two-fold reduction in their cost could make a big difference to the economics of renewable fuels and chemicals; Thermostability is a requirement of efficient cellulases, because at higher temperatures, 70 or even 80 degrees Celsius--chemical reactions are more rapid. In addition, cellulose swells at higher temperatures, which makes it easier to break down. Unfortunately, the known cellulases from nature typically won't function at temperatures higher than about 50°C. Cellulolytic anaerobic bacteria use macromolecular structures known as cellulosomes to hydrolyze recalcitrant cellulosic substrates [1,2]. Within the cellulosome, cellulases and other glycoside hydrolases [3,4] are assembled onto multidomain scaffoldin proteins for efficient degradation of cellulosic substrates [4]. Cellulosome assembly is achieved by binding dockerin domains from enzymes with cohesin domains in scaffoldin, while localization with substrate is mediated by one or more Carbohydrate Binding Modules (CBMs) on the scaffoldin [1,2,5]. The modularity of cellulosomes has spurred interest in ‘designer cellulosomes’ [6], where different cellulases are synthetically combined for a specific application. Within a given glycoside hydrolase family, a diverse pool of potential cellulases would be beneficial for designer cellulosomes by providing a suite of enzymes with differing properties and an extensive platform for further enzyme engineering. Family 48 cellulases (Cel48) are ideal candidates for designer cellulosomes [3]. As one of the most important families of bacterial cellulases, they are usually a major constituent of bacterial cellulosomes [4,7–12]. Of the 116 bacterial Cel48 genes currently predicted in the CAZy database (http://www.cazy.org/) only 13 have been characterized. We chose SCHEMA recombination to plan to synthesize a diverse set of new family 48 sequences. SCHEMA is a structure-guided, site-directed protein recombination method that has been used to generate thousands of novel P450s, β-lactamases, and fungal cellulases. The chimeric proteins that are made by recombining natural sequences differ. Our objective for this project was to construct chimeric synthetic cellulase genes for production of thermostable cellulases for efficient breakdown of cellulose at high temperature.

## Materials and Methods

Genomic DNA from bacteria Cellulomonas sp. (ATCC^®^ 21399) was used as a template to do PCR using standard PCR reagents and assay conditions using the primers:

**Table:** 

CCELcdCTHEdock+XbaIfwd	GCAATACTCTTCCCAGATTCTAGAATGACAT ATAAAGTACCTGGTACTCCTTCTACT
CCELcdCTHEdock+XbaIrev	AGGTACTTTATATGTCATTCTAGAATCTGGG AAGAGTATTGCATAAACTCCATTTGC

**Figure 1:**
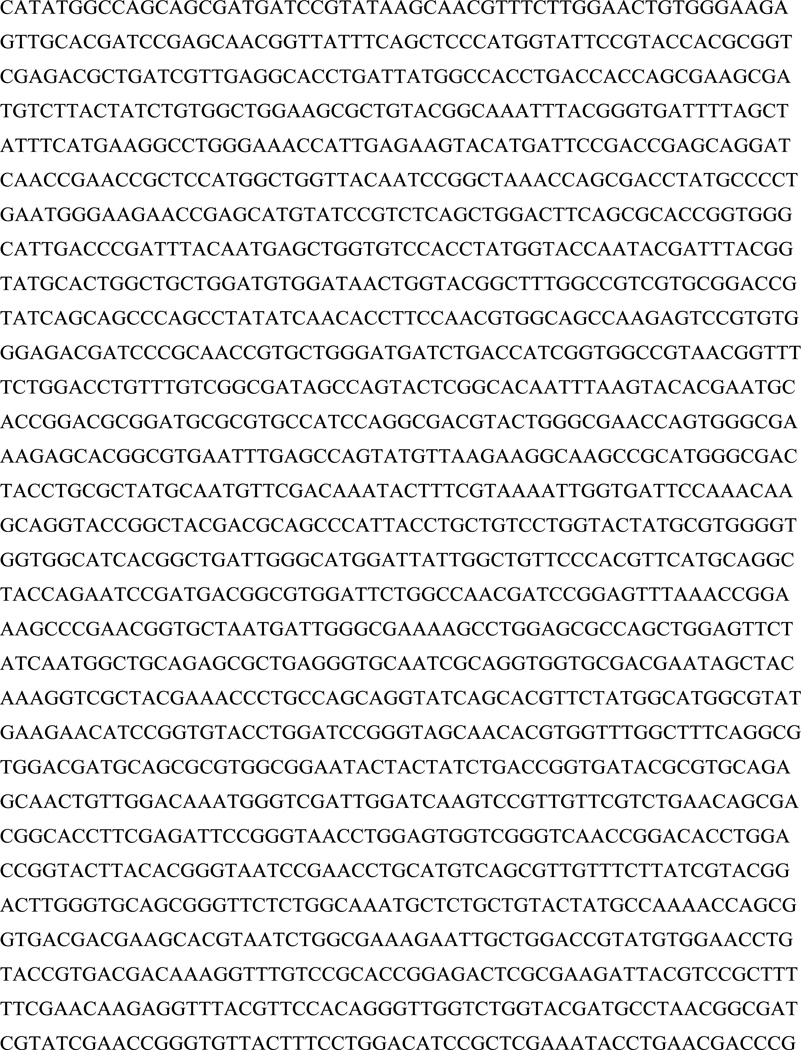
Nucleotide sequence of the PCR amplified amplicon.

**Figure 2:**
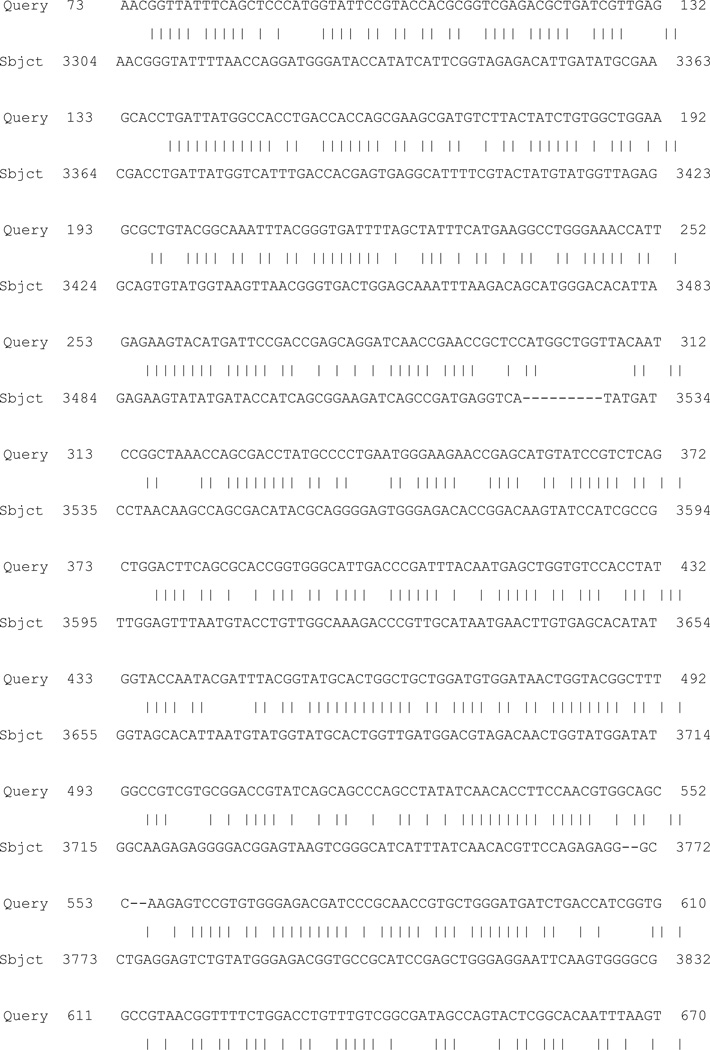

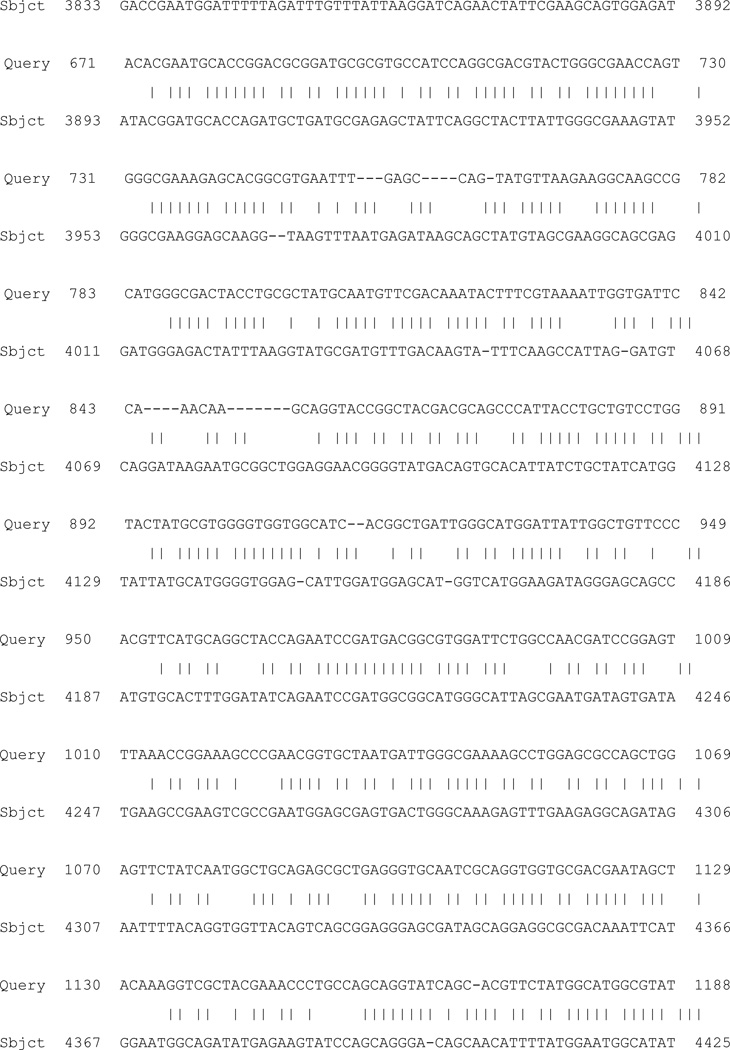

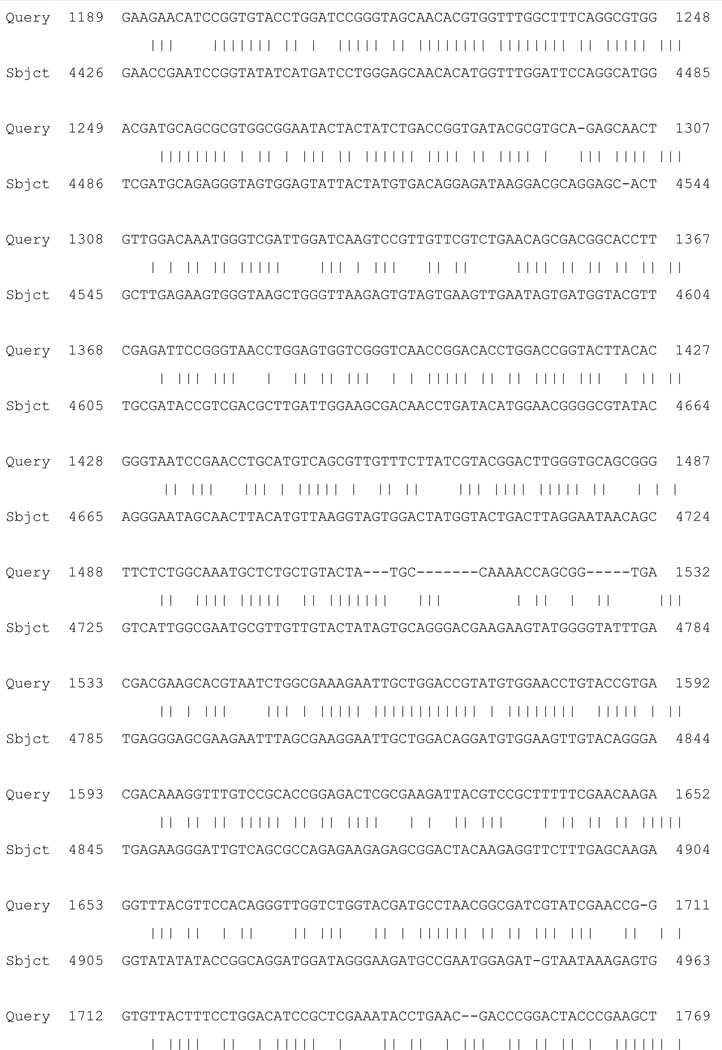

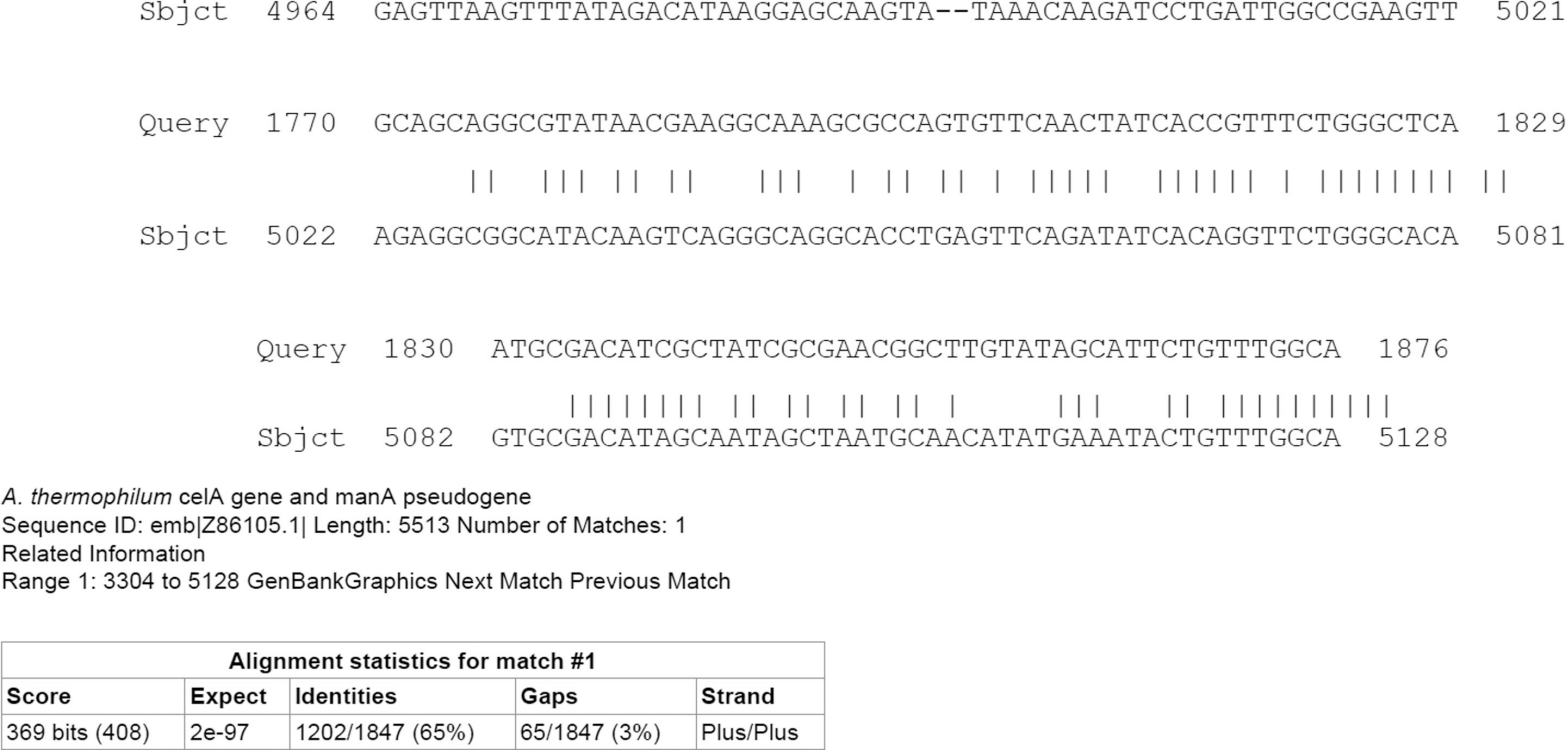
NCBI-BLAST search result of the sequenced amplicon DNA.

The amplicon was further sequenced and the obtained sequence (Figure 1) was subjected to NCBI-BLAST search and showed homology to *A. thermophillum* celA gene (Figure 2).

**Figure 3:**
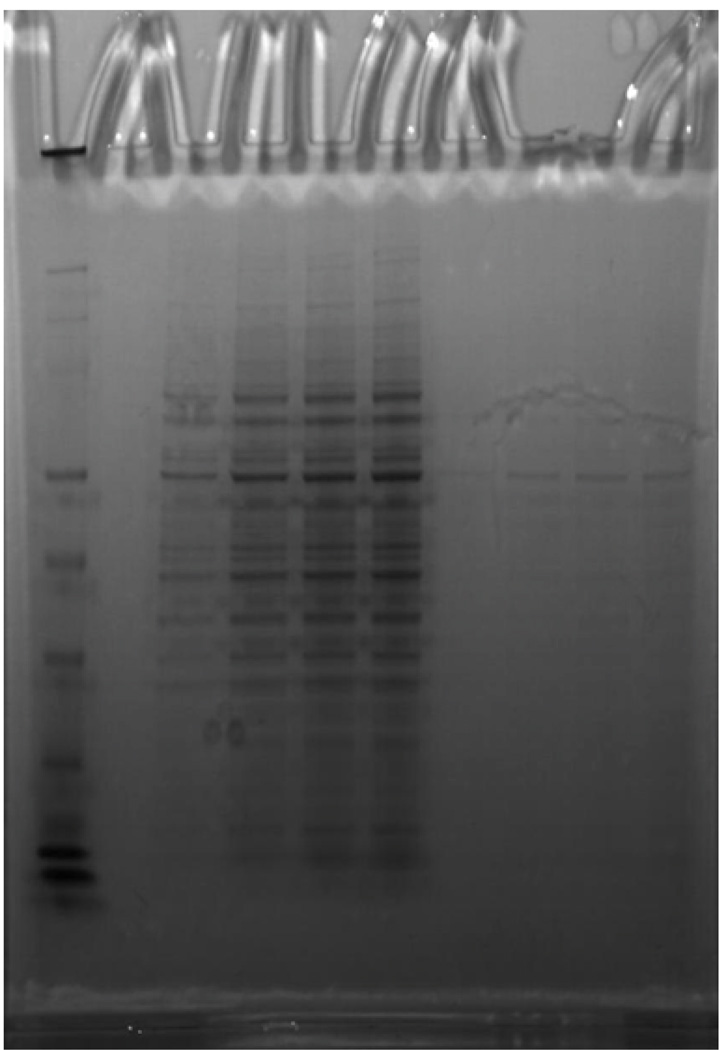
Lane 1=Protein marker, Lane 3–6=Different fractions of bacterial protein expressed, Lane 7–10=His-tag purified recombinant cellulase like Chimeric protein.

The amplicon was then cloned into a Gateway System (Invitrogen, USA) his-tag expression vector and BL-21 *E. coli* bacteria was transformed with this construct. The bacteria was then grown in LB medium and IPTG was used to induce the protein, which was then his-tag purified using a nickel column (please see the gel picture in Figure 3), protein concentration was measured by using standard Bradford method (Sigma, USA).

## Cellulase Assay

### Method

A standard assay for cellulase activity was performed with a reaction mixture containing 0.52% carboxymethyl cellulose in 10 mM sodium phosphate (pH 7.0) at 30°C. Reduced sugar produced by the reaction was determined using the method described by Park and Johnson [13] using a standard BioRad (USA) spectrophotometer.

## Results and Discussion

We were interested to synthesize a chimeric synthetic cellulase gene from the different cellulases DNA sequence that are there in the gene bank to produce a thermostable cellulose, our initial bioinformatics analysis by using the CAZy database and SCHEMA recombination to design gene sequences which will fulfill those conditions resulted in production of a chimeric protein. We derived the following full length DNA sequence (Figure 1) which showed homology to Cel A gene of *A. thermophillum* (Figure 2) and we expressed and purified the recombinant protein by His-tag method (Figure 3). The activity of this novel chimeric protein was determined to be cellulase when tested for activity by standard Park Johnson assay (Table 1). Thus our recombinant chimeric proteins have definite Cellulase enzyme characteristics. We look forward to scaling up productions and temperature and pH stability testing for its usefulness for bioremediation.

## Figures and Tables

**Table 1:** Showing cellulase bioactivity of the novel recombinant chimeric protein by Park Johnson Assay.

Enzyme Concentration	Bioactivity
100 µg/µl	0.50
50 µg/µl	0.25
25 µg/µl	0.15
10 µg/µl	0.05
